# The prevalence and mechanism of triclosan resistance in *Escherichia coli* isolated from urine samples in Wenzhou, China

**DOI:** 10.1186/s13756-020-00823-5

**Published:** 2020-10-02

**Authors:** Weiliang Zeng, Wenya Xu, Ye Xu, Wenli Liao, Yajie Zhao, Xiangkuo Zheng, Chunquan Xu, Tieli Zhou, Jianming Cao

**Affiliations:** 1 Department of Medical Laboratory Science, School of Laboratory Medicine and Life Science, Wenzhou Medical University, Wenzhou, Zhejiang Province China; 2 Department of Clinical Laboratory, The First Affiliated Hospital of Wenzhou Medical University, Wenzhou, Zhejiang Province China

**Keywords:** *Escherichia coli*, Triclosan, Efflux pump, Resistance, *fabI*, Cross-resistance, Multidrug-resistance

## Abstract

**Background:**

The widespread application of triclosan contributes to its residual deposition in urine, which provides an environment of long-term exposure to triclosan for the intestinal *Escherichia coli*. We determined the triclosan and antibiotic resistance characteristics of *E. coli* strains isolated from urine samples and further investigated the resistance mechanism and molecular epidemic characteristics of triclosan-resistant *E. coli* isolates.

**Methods:**

A total of 200 non-repetitive *E. coli* strains were isolated from urine samples and then identified. The minimum inhibitory concentrations (MICs) of triclosan and antibiotics, *fabI* mutation, efflux pump activity, the expression of 14 efflux pump encoding genes, and epidemiological characteristics were determined by the agar dilution method, polymerase chain reaction (PCR), carbonyl cyanide 3-chlorophenylhydrazone (CCCP) inhibition test, quantitative real-time polymerase chain reaction (RT-qPCR), multilocus sequence typing (MLST), and pulse-field gel electrophoresis (PFGE) for all triclosan-resistant isolates. Furthermore, we also investigated the effect of triclosan exposure in vitro on antibiotic susceptibility and the efflux pump encoding gene expressions of triclosan-susceptible strains via serial passage experiments.

**Results:**

Of the 200 *E. coli* isolates, 2.5% (*n* = 5) were found to be resistant to triclosan, and multidrug resistance (MDR) and cross-resistance phenotypes were noted for these triclosan-resistant strains. The triclosan-sensitive strains also exhibited MDR phenotypes, probably because of the high resistance rate to AMP, CIP, LVX, and GEN. Gly79Ala and Ala69Thr amino acid changes were observed in the triclosan-resistant strains, but these changes may not mediate resistance of *E. coli* to triclosan, because mutations of these two amino acids has also been detected in triclosan-susceptible strains. Moreover, except for DC8603, all other strains enhanced the efflux pumps activity. As compared with ATCC 25922, except for *fabI*, increased expressions were noted for all efflux pump encoding genes such as *ydcV*, *ydcU*, *ydcS*, *ydcT*, *cysP*, *yihV*, *acrB*, *acrD,* and *mdfA* among the studied strains with varying PFGE patterns and STs types. Unexpectedly, 5 susceptible *E. coli* isolates showed rapidly increasing triclosan resistance after exposure to triclosan in vitro for only 12 days, while MDR or cross-resistance phenotypes and the overexpression of efflux pump genes were recorded among these triclosan-induced resistant isolates.

**Conclusions:**

This is the first study to report that short-term triclosan exposure in vitro increases triclosan resistance in susceptible *E. coli* isolates. After acquiring resistance, these strains may present MDR or cross-resistance phenotypes. Moreover, triclosan resistance mainly involves the overexpression of *fabI* and efflux pumps in *E. coli* isolates.

## Background

*Escherichia coli* has been implicated in most hospital- and community-acquired infections, including several intestinal and extraintestinal infections, urinary tract infection, and some fatal infections that develop in immunocompromised patients [1–3]. Over the past few decades, self-medication and inappropriate usage of antibiotics has contributed to the increasing drug resistance in clinical practice. Even worse, the treatment of *E. coli* infections has become extremely challenging because of the increasing multidrug-resistance to antibiotics [4, 5].

Triclosan, which is a broad-spectrum and highly effective antibacterial agent, can inhibit various microorganisms even at low concentrations, and be bactericidal at high concentrations [6]. In fact, it is also used for disinfecting medical equipment to prevent possible contamination [7]. Hence, triclosan plays a key role in reducing the dissemination and the spread of pathogenic bacteria across hospital and community environments.

Unfortunately, owing to its increased clinical application, the obvious levels of triclosan in various natural and engineered environments, such as soil and water, even in the body fluids of babies and adults have been reported [8–10]. Yin et al. have estimated that the average concentration of triclosan is presently 0.36 μg/L in 80% of the urine samples in China [11]. Furthermore, triclosan is considered as a “new environmental endocrine disruptor” because of its potential endocrine disrupting effects, which had an adverse effect on the human health [12]. Unfortunately, a recent study demonstrated that triclosan can spread antibiotic-resistance genes [13]. In other words, it is presumed that the long-term use of triclosan would contribute to the deposition of triclosan residue in the human urine. In addition, the widespread use of triclosan on bacterial resistance has also been suggested. As both these aspects are believed to affect the human health, but remain controversial, it is essential to gather more evidences about the effect of triclosan on drug resistance in *E. coli* isolated from urine samples [14].

Long-term exposure to triclosan promotes reduced sensitivity to triclosan in *E. coli* through extensive resistance mechanisms in vitro [15]. Of which, active efflux is a resistance mechanism that involves reducing the drug concentration in bacteria, whereby the efflux system pumps the intracellular antibacterial drugs out of the cell; this event confers the bacteria with resistance against a wide range of antimicrobials and biocides, including triclosan [16, 17]. Indeed, several drug efflux pumps are known to mediate resistance to traditional antibiotics and biocides, including the resistance nodulation division (RND) family, the major facilitator superfamily (MFS), the small multi-resistance (SMR), and the multidrug and toxic compound extrusion (MATE) families [18]. In addition, *fabI* mutation also contributes to *E. coli* resistance to triclosan [19]. However, the role of different types of efflux pumps is not well understood in triclosan-resistant *E. coli* isolated from urine samples.

It is important to conduct further research to yield a better scientific theoretical basis for the rational use of triclosan and toward nosocomial infection control. Our study describes the resistance profile of *E. coli* isolated from urine samples, warranting further investigation of the action mechanism of triclosan as well as its molecular epidemiology characteristics.

## Methods

### Bacterial strains and identification

A total of 200 non-repetitive *E. coli* strains isolated from the urine samples of urinary tract infection (UTI) patients admitted to the Affiliated Hospital of Wenzhou Medical University in Wenzhou, China in 2018 were collected. The isolates were identified by using the Matrix-Assisted Laser Desorption/Ionization Time of Flight Mass Spectrometry (MALDI-TOF MS; bioMérieux, Lyons, France).

### Minimum inhibitory concentrations of triclosan

We measured the MICs of triclosan in accordance with a previous study; isolates with MICs ≥MIC_90_ (the concentration required to inhibit growth by 90% isolates; MIC_90_ = 0.5 μg/mL) were considered to be resistant [20]. *E. coli* ATCC 25922 was used as the quality control strain.

### Antimicrobial susceptibility test

A total of 200 *E. coli* isolates were subjected to antimicrobial susceptibility testing for 10 clinical conventional antibiotics by the agar dilution method, such as ampicillin (AMP), ciprofloxacin (CIP), levofloxacin (LVX), cefepime (FEP), ceftazidime (CAZ), ertapenem (ETP), imipenem (IPM), gentamicin (GEN), nitrofurantoin (NIT), and tobramycin (TOB). Our results were interpreted by the latest guidelines from the Clinical and Laboratory Standards Institute (CLSI).

### Detection of *fabI* mutation by PCR

Genome DNA of triclosan-resistant *E. coli* strains as well as randomly selected equal numbers of triclosan-susceptible strains were extracted by using the Biospin Bacterial Genomic DNA Extraction Kit (Bioflux, Tokyo, Japan) in accordance with the manufacturer’s instructions. Then, *fabI* was amplified by PCR with specific oligonucleotide primers, and the positive PCR products were directly sequenced by the Shanghai Genomics Institute Technology Co. Ltd. [17]. Next, gene mutations were further analyzed according to the GenBank accession number NC000913.3 of the *E. coli* genome assembly used in the BLAST (https://blast.ncbi.nlm.nih.gov/Blast.cgi) comparisons [21]. Moreover, 14 known drug efflux pump encoding genes (*ydcT*, *ydcU*, *ydcV*, *ydcS*, *cysP*, *cysU*, *marA*, *soxS*, *yhiv*, *acrB*, *acrD*, *acrF*, *mdfA*, and *norE*) were also amplified with the RT-qPCR primers in order to ensure that the strain carried the gene for subsequent RT-qPCR experiments. The PCR and RT-qPCR primers used are listed in the Supplementary Table S1 (see Additional file 1).

### Efflux pump inhibition test

To test the efflux pump activity of triclosan-resistant *E. coli* strains, efflux pump inhibitor CCCP was tested. The resistant strains were tested on agar plates without or with 10 μg/mL CCCP by the agar dilution method. Compared with for triclosan alone, the MICs value of triclosan combination with 10 μg/mL CCCP decreased to ≥4, which confirmed a positive inhibitory effect [22]. In addition, the concentration of 10 μg/mL was determined as the optimal sub-minimum inhibitory concentrations (sub-MICs) that could inhibit the overexpression of efflux pump without affecting the growth of bacteria using the agar dilution method.

### Expression levels of efflux pumps by RT- qPCR

In addition to detecting the *fabI* expression, 14 efflux pump encoding genes were also examined by RT-qPCR, which included the ABC transporters system encoding the genes *ydcT*, *ydcU*, *ydcV*, *ydcS*, *cysP*, and *cysU*; the Arac-regulator genes *marA* and *soxS*; the RND efflux pump encoding genes *yhiv* and *acrBDF*; the MdfA efflux TolC encoding genes *mdfA*; and the NorE efflux pump encoding gene *norE*.

Briefly, triclosan-resistant strains with an active efflux pump were also tested, while ATCC 25922 served as the control strain. The abovementioned strains were also inoculated in fresh Luria broth (LB) and allowed to grow to the logarithmic phase (OD_600_ = 0.6). The total cellular RNA of these cultures was extracted by using the Bacterial RNA Miniprep Kit (Biomiga, Shanghai, China) according to the manufacturer’s recommendation. Subsequently, the purified RNA was reverse transcribed into cDNA via the RevertAid First Strand cDNA Synthesis Kit (Thermo Scientific, MA, USA) and amplified by using the TB Green Premix Ex Taq II (Tli RNaseH Plus) (2×) (Takara, Japan). In the PCR reaction, a global gene *gapA* and the housekeeping gene *16S rRNA* were used as the corresponding internal controls, and the quantification of efflux pump genes was performed by the 2^˗ΔΔCt^ method. An expression of ≥2 in comparison with that of the control strain ATCC 25922 indicated an upregulation, which is in accordance with a previous report [21]. Specific RT-qPCR primers are listed in the Supplementary Table S1 (see Additional file 1). All experiments were performed in at least 3 biological replicates, and the data were expressed as the mean ± SD (Supplementary Table S2; see Additional file 1).

### Genotyping by MLST

All triclosan non-susceptible isolates were typed using the MLST method. The sequences of 8 housekeeping genes (*trpB*, *uidA*, *dinB*, *icdA*, *pabB*, *polB*, *put*, and *trpA*) were amplified with specific primers available at the MLST database (https://bigsdb.pasteur.fr/index.html), and the sequence types (STs) were evaluated in comparison with the allelic profiles to the MLST database [23].

### Strain-typing PFGE

To confirm and analyze the clonal relatedness among the triclosan-resistant isolates, PFGE was performed in accordance with the PulseNet protocols published by the US Centers for Disease Control and Prevention (CDC) with some minor modifications. Briefly, the cell suspensions were treated with protease K and incubated with the XbaI restriction enzyme for at least 2 h at 37 °C to digest the DNA fragments. Then, PFGE was performed using the CHEF-MAPPER XA PFG system (Bio-Rad, USA) for 18 h. The detailed running condition were as follows: initial switch time value of 2.16 s and a final switch time of 54.17 s at a gradient of 6 V/cm at a 120° included angle [24]. Next, the electrophoretic banding patterns were visualized by the GelDoc XR gel imaging system (Bio-Rad, USA) and further analyzed by Quantity One (Bio-Rad Laboratories, USA). The Unweighted Pair Group Method with Arithmatic Mean (UPGMA) with optimization set at 1.5% to create the dendrogram at the cut-off line ≥85% was considered to analyze the genetic relatedness [25]. The standard *Salmonella* strain H9812 was considered as the positive control.

### Serial passage experiment

In order to determine whether triclosan exposure in vitro increased the bacterial resistance, as previously described, serial passage experiment was conducted for triclosan-susceptible isolates DC8361, DC8363, DC8400, DC8413, and DC8510 [26]. Specifically, the isolates were cultivated on Macconkey agar plate and cultured overnight at 37 °C to obtain a single isogenic strain, which was then inoculated into 3-mL fresh LB broth with different concentrations of triclosan at 37 °C for overnight, and the ticlosan gradient concentrations were 0.0625, 0.125, 0.25, 1, 2, 4, 8, 16, 32, 64, and 128 μg/mL. Culture supernatants with bacterial growth in the highest triclosan concentrations were aspirated and continuously passaged in fresh triclosan gradients, and after only 12 days of triclosan exposure, triclosan-mutant strains with MICs ≥32 μg/mL were obtained.

Next, the stability of triclosan resistance was confirmed via continuous passage in vitro. Briefly, the triclosan-mutant strains were cultured in 3-mL fresh LB broth without triclosan at 37 °C for 24 h. Every 24 h, 30 μL of overnight culture supernatants were transferred to another 5-mL tube containing 2.97-mL of fresh LB broth without triclosan. After 12 days, the MICs of triclosan and antibiotics and the expression levels of efflux pump genes were tested in triplicate, respectively, using the same method described previously.

## Results

### The MICs of triclosan and antibiotics

**Fig. 1:**
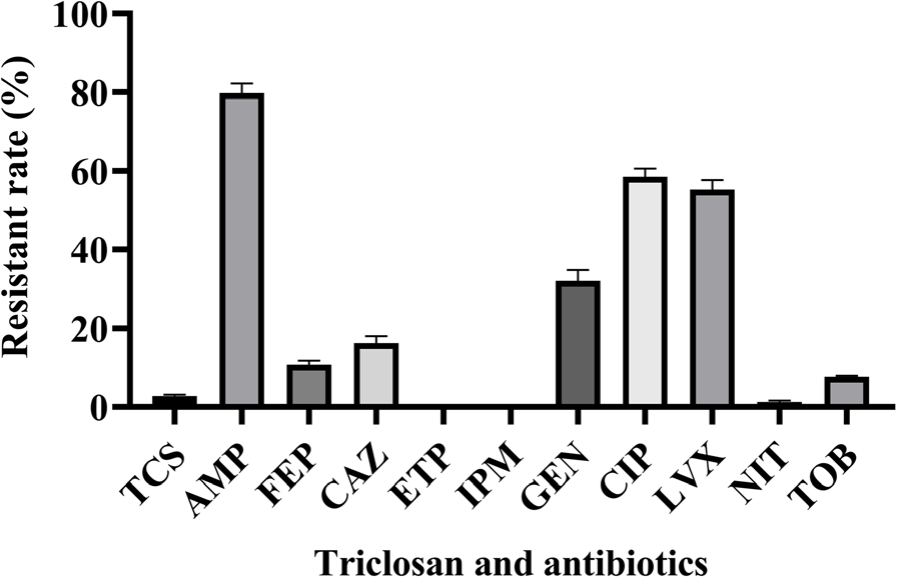
Triclosan susceptibility and antimicrobial resistance profile. TCS, triclosan; AMP, ampicillin; CIP, ciprofloxacin; LVX, levofloxacin; FEP, cefepime; CAZ, ceftazidime; ETP, ertapenem; IPM, imipenem; GEN, gentamicin; NIT, nitrofurantoin; TOB, tobramycin

Only 5 triclosan-resistant isolates were selected (2.5%, 5/200), with triclosan MICs 0.03125–8 μg/mL, and a low triclosan-tolerance rate of *E. coli* was recorded from the urine specimens (Table 1). Interestingly, these triclosan-resistant isolates tended to be resistant to multiple antibacterial agents, including AMP, FEP, CAZ, and GEN. The resistance profiles shown in Fig. 1, all 200 *E. coli* isolates showed susceptibility to ETP and IPM, and most of the *E. coli* isolates were also susceptible to FEP, CAZ, NIT, and TOB, but resistant to AMP, CIP, LVX, and GEN, with the resistance rate of 81.5, 57, 53.5, and 34%, respectively, suggesting that triclosan-sensitive strains may also exhibit multiple-resistant phenotypes. To explore the relationship between triclosan exposure and MDR phenotypes, further serial passage experiment are warranted.

**Table 1:** Mutations of *fabI* and MICs of triclosan and antibiotics against *E. coli* strains

Isolates	Triclosan MICs (μg/mL)	Antibiotic MICs ^a^ (μg/mL)										Mutations in *fabI* ^b^
		AMP	CIP	LVX	FEP	CAZ	ETP	IPM	GEN	NIT	TOB	
DC8358	8	**> 128**^**c**^	0.5	0.5	**32**	**16**	**16**	2	**> 64**	64	**> 64**	ND ^d^
DC8419	4	**> 128**	**> 32**	**64**	**16**	**16**	< 0.5	< 0.25	**> 64**	32	**32**	Gly79Ala
DC8424	4	**> 128**	**> 32**	**16**	**> 64**	**> 64**	**> 32**	**> 32**	**> 64**	64	**> 64**	Ala69Thr
DC8603	2	**> 128**	**> 32**	**> 64**	**64**	**32**	< 0.5	< 0.25	< 2	**> 128**	< 2	ND
DC8724	8	**> 128**	**> 32**	**32**	**32**	**64**	**16**	1	**> 64**	16	**> 64**	ND
DC8361	0.125	< 4	< 0.25	0.5	< 1	< 2	< 0.5	< 0.25	< 2	16	< 2	Met2Arg; Val5Phe; Ala69Thr
DC8363	0.125	**> 128**	< 0.25	0.5	2	< 2	< 0.5	< 0.25	< 2	< 8	< 2	Ser5Leu; Gly79Ala
DC8400	0.125	**> 128**	< 0.25	< 0.25	< 1	< 2	< 0.5	< 0.25	< 2	16	< 2	Val4Ser; Ala69Thr
DC8413	0.25	**> 128**	< 0.25	< 0.25	< 1	< 2	< 0.5	< 0.25	< 2	< 8	< 2	Gly79Ala
DC8510	0.125	**> 128**	< 0.25	< 0.25	2	< 2	< 0.5	< 0.25	< 2	< 8	< 2	Gly79Ala; Asp 235Glu

^a^ *MICs* Minimum inhibitory concentration. *AMP* ampicillin, *CIP* ciprofloxacin, *LVX* levofloxacin, *FEP* cefepime, *CAZ* ceftazidime, *ETP* ertapenem, *IPM* imipenem, *GEN* gentamicin, *NIT* nitrofurantoin, *TOB* tobramycin. ^b^ *Gly* Glicine, *Ala* Alanine, *Thr* Threonine, *Met* Methionine, *Arg* Arginine, *Val* Valine, *Phe* Phenylalanine, *Ser* Serine, *Leu* Leucine, *Asp* aspartic acid, *Glu* glutamic acid. ^c^ The values in bold font indicate resistance. ^d^ *ND* Not detected

### Triclosan-induced isolates with reduced susceptibility to triclosan presented MDR or cross-resistance phenotypes after short-term triclosan exposure in vitro

Stable triclosan-induced strains with reduced susceptibility to triclosan were grown under triclosan pressure via serial passaging. The MICs of triclosan and the tested antibiotics for the parent and induced strains are shown in Table 2. After exposure to a subinhibitory concentration of triclosan in vitro for only 12 days, the derived triclosan-resistant strains emerged immediately, and the MICs of triclosan increased by 256–512 times. Notably, triclosan-induced strains showed a slight decrease in susceptibility to IPM and NIT, but an obvious increase in resistance to CIP, LVX, FEP, and TOB. In a word, each mutant displayed intermediate or high level of resistance to at least one antibiotic, suggesting that these triclosan-resistant mutants had MDR or cross-resistance phenotypes.

**Table 2:** MICs of triclosan and antibiotics against the parent and mutant *E. coli* strains

Isolates	DC8361		DC8363		DC8400		DC8413		DC8510	
	Pre^a^	Post^b^	Pre	Post	Pre	Post	Pre	Post	Pre	Post
TCS^c^	0.125	**32**	0.125	**32**	0.125	**64**	0.25	**128**	0.125	**32**
AMP	< 4	< 4	**>128**^**d**^	**> 128**	**> 128**	**> 128**	**> 128**	**> 128**	**> 128**	**> 128**
CIP	< 0.25	< 0.25	< 0.25	< 0.25	< 0.25	**1**	< 0.25	**2**	< 0.25	< 0.25
LVX	0.5	0.5	0.5	0.5	< 0.25	**2**	< 0.25	**1**^**e**^	< 0.25	< 0.25
FEP	< 1	< 1	2	**4**^**e**^	< 1	**4**^**e**^	< 1	< 1	2	2
CAZ	< 2	< 2	< 2	< 2	< 2	< 2	< 2	< 2	< 2	< 2
ETP	< 0.5	< 0.5	< 0.5	< 0.5	< 0.5	< 0.5	< 0.5	< 0.5	< 0.5	< 0.5
IPM	< 0.25	< 0.25	< 0.25	< 0.25	< 0.25	1	< 0.25	< 0.25	< 0.25	1
GEN	< 2	< 2	< 2	< 2	< 2	< 2	< 2	< 2	< 2	< 2
NIT	16	32	< 8	32	16	16	< 8	16	< 8	16
TOB	< 2	**8**^**e**^	< 2	< 2	< 2	< 2	< 2	**8**^**e**^	< 2	**8**^**e**^

^a^ *Pre* wild-type strains; ^b^ *Post* mutant strains; ^c^ *MICs* Minimum inhibitory concentration, *TCS* triclosan, *AMP* ampicillin, *CIP* ciprofloxacin, *LVX* levofloxacin, *FEP* cefepime, *CAZ* ceftazidime, *ETP* ertapenem, *IPM* imipenem, *GEN* gentamicin, *NIT* nitrofurantoin, *TOB* tobramycin, ^d^ The values in bold font indicates resistance; ^e^intermediate

### Analysis of *fabI* mutation

PCR revealed that the *fabI* and 14 efflux pump encoding genes were present in all of the tested strains, except for the *acrF*. A variety of different mutations were detected in both the resistant and susceptible strains. In DC8419 and DC8424 strains, the Gly79Ala and Ala69Thr mutations were detected, respectively. However, these mutations were also detected in the susceptible strains. In addition, we discovered other mutations of *fabI* in the susceptible strains, such as Met2Arg, Ser5Leu, Val4Ser, and Asp235Glu (Table 1).

### Efflux pump phenotype test

We sought to further investigate the triclosan-resistance mechanism among the resistant isolates. In compared to the absence of CCCP, the triclosan MICs of DC8358, DC8419, DC8424, and DC8724 reduced by 8, 4, 4, and 16 times in the presence of 10 μg/mL CCCP, respectively. Our results indicated that the efflux pumps systems were extremely active among the abovementioned 4 isolates. Inversely, DC8603, unlike other resistant strains, displayed a negative phenotype in the efflux pump test, which remained unaffected by the MICs of triclosan without or with CCCP (Table 3).

**Table 3:** Efflux pump phenotype test

Isolates	MICs (μg/mL)		fold changes	Efflux pump phenotype^a^
	Triclosan	Triclosan + CCCP (10 μg/mL)		
DC8358	8	1	8	+
DC8419	4	1	4	+
DC8424	4	1	4	+
DC8603	2	2	1	–
DC8724	8	0.5	16	+

^a^Compared with triclosan alone, the MICs value of triclosan decreased ≥4 was confirmed to have an inhibitory effect when triclosan was used in combination with 10 μg/mL CCCP. ^+^indicates the strains with positive efflux pump phenotype. ^−^indicates the strains with negative efflux pump phenotype

### Expression levels of *fabI* and efflux pump encoding genes

**Fig. 2:**
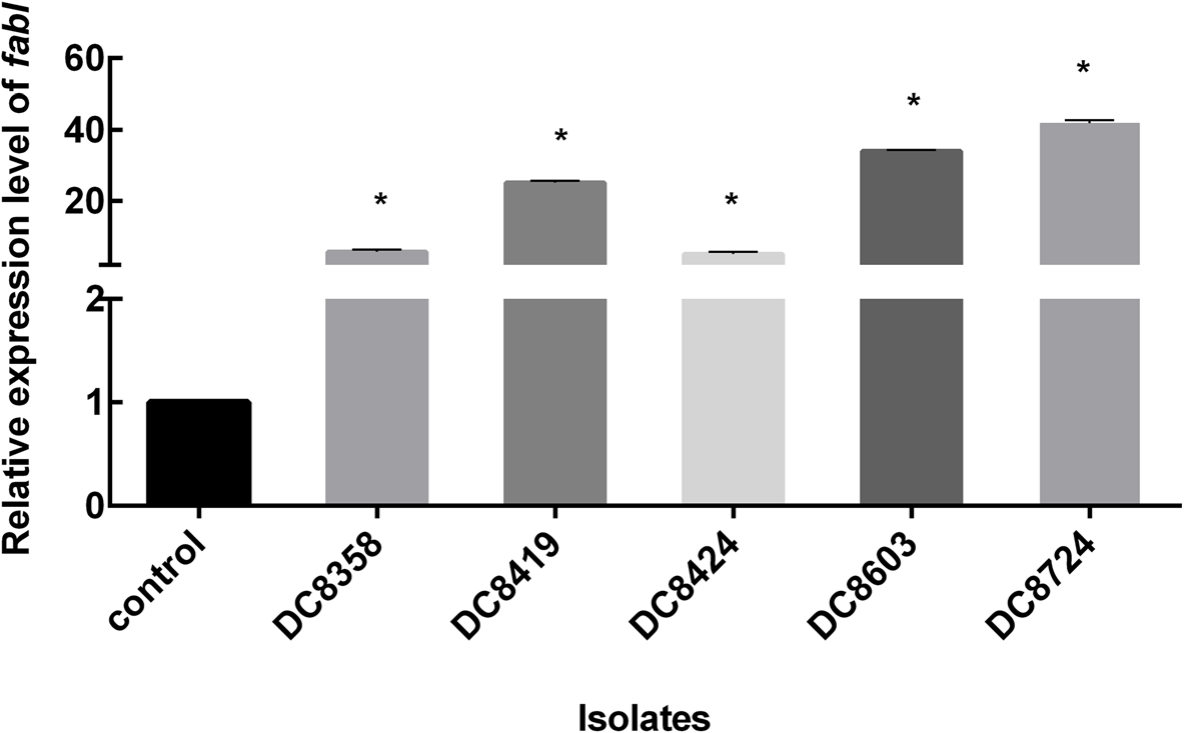
Relative *fabI* expression level. The values of three biological repeats represent the mean ± SD. *indicates the gene overexpression, which is the relative expression level increased by 2-fold or greater in comparison with that of the control strain *E. coli* ATCC 25922. In comparison to ATCC 25922, the *fabI* expression was increased (> 2-fold) in DC8358 (fold-changes: 5.69 ± 0.49), DC8419 (fold-changes: 25.14 ± 0.42), DC8424 (fold-changes: 5.11 ± 0.43), DC8603 (fold-changes: 34.05 ± 0.23), and DC8724 (fold-changes: 41.85 ± 0.59)

The assessment of the expression levels of *fabI* in this study revealed > 2-fold increased expression of *fabI* in all triclosan-resistant strains. In comparison with the triclosan-susceptible control strain ATCC 25922, the 5.69–41.85-fold-changes were noted for the *fabI* expression (Fig. 2).

**Fig. 3:**
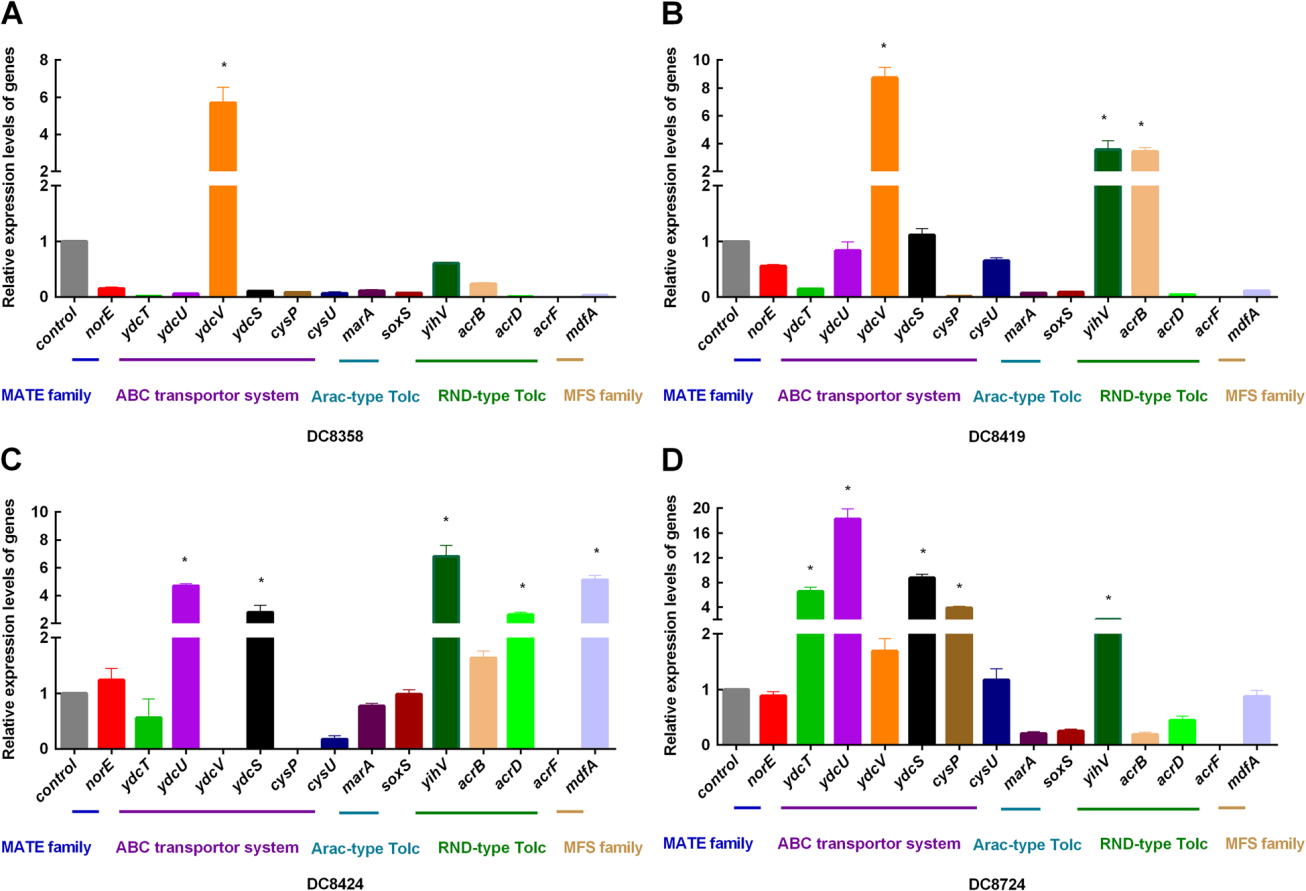
Relative gene expression levels. **a** The relative expression levels of efflux pump encoding genes in DC8358, the expression of *ydcV* (fold-changes: *ydcV*, 5.71 ± 0.68) was increased; **b** The relative expression levels of efflux pump encoding genes in DC8419, the expression of *ydcV*, *yihV*, and *acrB* (fold-changes: *ydcV*, 8.74 ± 0.61; *yihV*, 3.57 ± 0.52; *acrB*, 3.44 ± 0.21, respectively) was increased; **c** The relative expression levels of efflux pump encoding genes in DC8424, the expression of *ydcU*, *ydcS*, *yihV*, *acrD*, and *mdfA* (fold-changes: *ydcU*, 4.71 ± 0.13; *ydcS*, 2.8 ± 0.42; *yihV*, 6.82 ± 0.65; *acrD*, 2.63 ± 0.14; *mdfA*, 5.13 ± 0.26, respectively) was increased; **d** The relative expression levels of efflux pump encoding genes in DC8724, the expression of *ydcT*, *ydcU*, *ydcS*, *cysP*, and *yihV* (fold-changes: *ydcT*, 6.56 ± 0.56; *ydcU*, 18.25 ± 1.36; *ydcS*, 8.76 ± 0.49; *cysP*, 3.89 ± 0.2; *yihV*, 2.00 ± 0.03, respectively) was increased. The values of three biological repeats represent the mean ± SD. *indicates the gene overexpression, which is the relative expression level increased by 2-fold or greater in comparison to that of the control strain ATCC 25922

In addition, to better comprehend the relationship between triclosan resistance and the expression levels of efflux pump genes, different efflux pump types were also tested (Fig. 3; Supplementary Table S2 (see Additional file 1)). Compared to that in *E. coli* ATCC 25922, the *ydcV* expression was evidently increased (> 2-fold) in DC8358 (fold-changes: *ydcV*, 5.71 ± 0.68). Enhanced expressions of *ydcV*, *yihV*, and *acrB* (fold-changes: *ydcV*, 8.74 ± 0.61; *yihV*, 3.57 ± 0.52; *acrB*, 3.44 ± 0.21, respectively) were observed for DC8419. Similarly, the expressions of *ydcU*, *ydcS*, *yihV*, *acrD*, and *mdfA* (fold-changes: *ydcU*, 4.71 ± 0.13; *ydcS*, 2.8 ± 0.42; *yihV*, 6.82 ± 0.65; *acrD*, 2.63 ± 0.14; *mdfA*, 5.13 ± 0.26, respectively) were increased for DC8424. The upregulation of active efflux pump genes *ydcT*, *ydcU*, *ydcS*, *cysP*, and *yihV* (fold-changes: *ydcT*, 6.56 ± 0.56; *ydcU*, 18.25 ± 1.36; *ydcS*, 8.76 ± 0.49; *cysP*, 3.89 ± 0.2; *yihV*, 2.00 ± 0.03, respectively) was noted for DC8724. These results were consistent with those of the efflux pump inhibition test, which indicated that efflux pumps overactivity could induce the overexpression of efflux pump genes, which in turn mediates triclosan resistance in *E. coli* isolates.

### Overexpression of efflux pump encoding gene mediated triclosan resistance in *E. coli* isolates

**Fig. 4:**
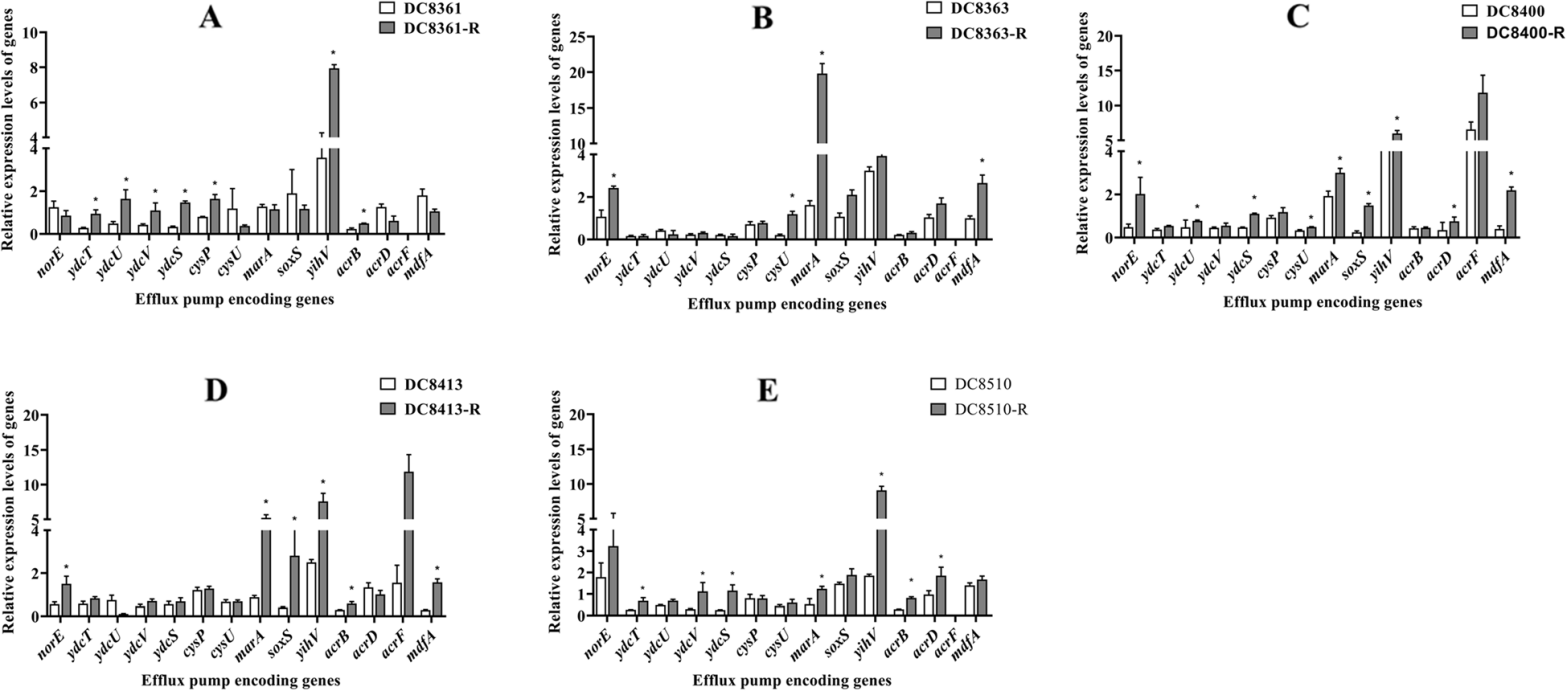
Relative gene expression levels in the parent strains and the induced strains. **a-e** The expression levels of 14 efflux pump encoding genes among DC8361 and DC8361-R, DC8363 and DC8363-R, DC8400 and DC8400-R, DC8413 and DC8413-R, and DC8510 and DC8510-R. -R indicates triclosan-induced strains; *indicates the relative expression levels increased by 2-fold or greater in comparison with those of the parent strains. The values of three biological repeats represent the mean ± SD

To better comprehend the role of efflux pumps in triclosan resistance of *E. coli* isolates, the expression levels of 14 efflux pump genes in 5 triclosan-susceptible strains before and after repeated exposure to sublethal concentrations of triclosan were also examined (Fig. 4; Supplementary Table S3 (see Additional file 1)). At least twice-fold increase in the efflux pump encoding genes in comparison to that for the parent strain without triclosan exposure indicated overexpression [21]. Based on our results, a significant increase in the expression of the efflux pump genes was noted in 80% of the induced isolates for *marA* and *yihV*, 60% of the induced isolates for *norE*, *ydcS*, *acrB*, and *mdfA;* 40% of the induced isolates for *ydcT*, *ydcU*, *ydcV*, *cysU*, *soxS*, and *acrD*, 20% of the induced isolates for *cysP*, and 0% of the induced isolates for *acrF*.

### Molecular epidemiological analysis

**Fig. 5:**
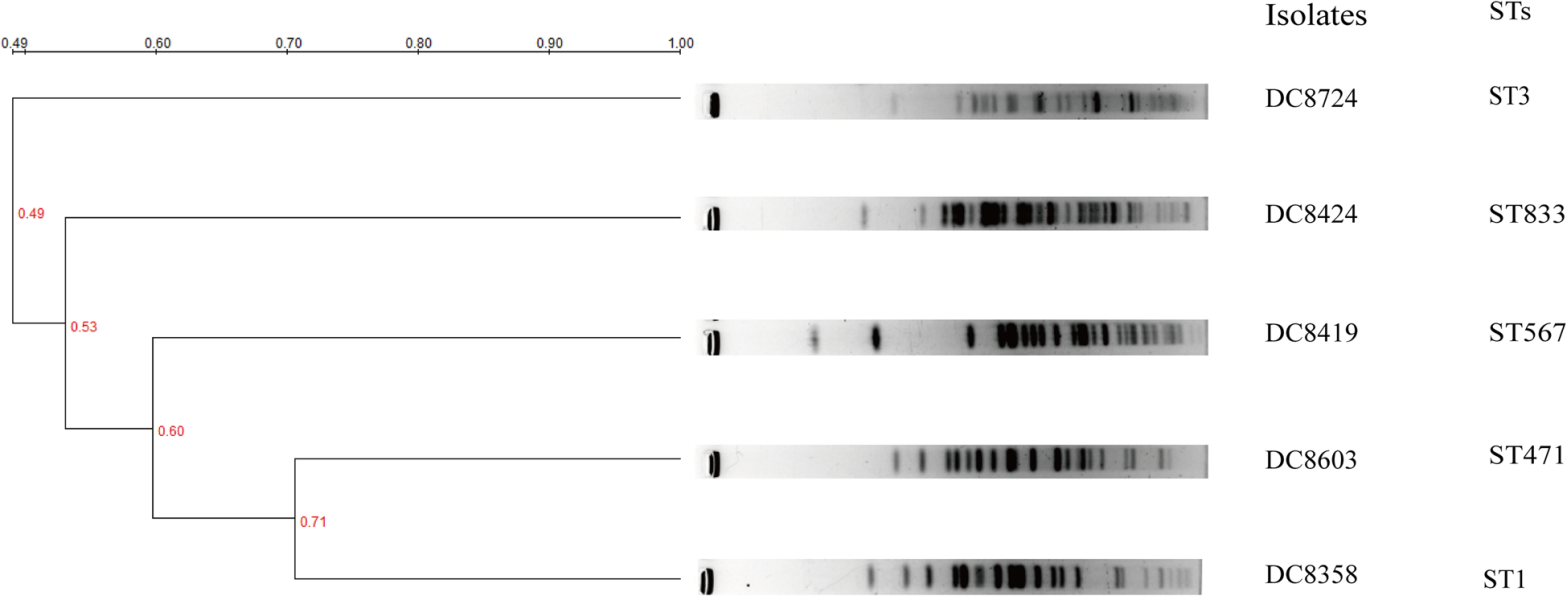
PFGE and MLST profiles of triclosan-resistant *E. coli* isolates. The relatedness of PFGE results was analyzed using the QualityOne software (Bio-Rad Laboratories, USA), and the phylogenetic tree was generated using the UPGMA clustering. The cut-off line ≥85% was considered to analyze genetic relatedness. The result show differences in the PFGE patterns and STs typing

PFGE analysis revealed that the similarity among these isolates was low (< 0.85) owing to the large differences in their PFGE patterns. Similarly, the MLST results confirmed their categorization into multiple and scattered STs, such as ST3, ST833, ST567, ST471, and ST1 (Fig. 5). Thus, the abovementioned results demonstrate that triclosan-resistant strains showed extremely low clonal relatedness in this study.

## Discussion

The usage of disinfectants has become common in clinics and household. As a result, increasing evidences have shown the presence of the disinfecting agent, for example, triclosan in human body fluids, such as urine, which has raised health concerns. The consensus on the relationship between biocides and antimicrobial resistance is controversial, and the effect of triclosan exposure on bacterial resistance is also unestablished.

In this study, we selected 5 triclosan-resistant strains (2.5%, 5/200) from 200 *E. coli* strains isolated from urine samples, and observed lower resistance rates among them in comparison to previous reports; these triclosan-resistant strains were characterized by their MDR profiles [20]. However, triclosan-sensitive clinical strains showed high rate of resistance to AMP, CIP, LVX, and GEN, which may also exhibit MDR phenotype. Obviously, triclosan-resistant strains were resistant to at least 6 types of antibiotics, indicating that the multidrug resistance rate of these strains was significantly higher than that of triclosan-sensitive strains. This fact raises the question of whether triclosan exposure affects or promotes bacterial resistance.

In our further research, we randomly selected 5 strains (DC8361, DC8363, DC8400, DC8413, and DC8510) that were sensitive to triclosan and to almost all antibiotics used in serial passage experiments, which proved that resistance developed in the selected isolates in only 12 days of triclosan exposure in vitro. In fact, MDR or cross-resistance phenotypes were also observed in these triclosan-induced resistant isolates. Consistent with previous reports, our results also suggested that triclosan exposure could rapidly increase bacterial resistance to triclosan, which was accompanied by a decrease in susceptibility to clinically important antimicrobial agents, such as CIP and LVX, owing to cross-resistance [27, 28]. One reasonable explanation for the mechanisms of cross-resistance between triclosan and antibiotics is that triclosan inhibits a specific bacterial target in a manner similar to that of clinically relevant antibiotics. Hence, it is possible that resistance to triclosan can confer cross-resistance to other antimicrobial agents via gene mutation and efflux pump activities [29]. However, the specific mechanisms of cross-resistance between triclosan and antibiotics need further research for clarification.

Based on the obtained phenomena and previous reports, we believe that triclosan exposure contributes not only to the acquisition of triclosan resistance but also facilitates the reduction in the susceptibility to clinical antimicrobials. This notion provides sufficient evidence for avoidance of the long-term use of triclosan in clinical practice [21, 27]. Moreover, it is worth noting that significantly reduced triclosan sensitivity occurred during the serial passage experiment, which provides a meaningful guidance for the reasonable use of triclosan, including regulation of its concentration and dosage, toward the prevention of increased resistance of pathogens to triclosan.

A past study suggested that effective upregulation of the efflux pump genes plays an important role in non-susceptibility of bacteria to biocides [30]. Accordingly, we tested the activity of 4 different types of TolC and their relative expressions. Under triclosan stimulation, increased efflux pump activity of the isolates were recorded in our study. In addition, the expression levels of ABC transporters system encoding genes *ydcT*, *ydcU*, *ydcV*, *ydcS*, and *cysU* and those of RND-type TolC encoding genes *acrB*, *acrD* and *yihV*, and *mdfA*, which belongs to the MFS family, was significantly increased in comparison with those of ATCC 25922; these findings are consistent with those of a previous study conducted in China [31]. In contrast to past reports, no increase in the expression levels of other efflux pump encoding genes was noted, probably indicating that ABC transport efflux pump, RND-type TolC, and MFS family activity are the relatively stronger advantages during the process of adaptive changes of the studied isolates to triclosan [17, 21]. Cumulatively, our results suggest a strong relationship between genes overexpression and increased tolerance of *E. coli* against triclosan, which suggests that multiple efflux pumps may synergistically mediate triclosan resistance. These findings were fufrther confirmed through serial passage experiments, wherein the expression levels of efflux pump encoding genes among triclosan-induced strains were found to be significantly higher than those of the parent strains. In conclusion, our results suggest that the overexpression of different efflux pump mediated triclosan resistance in different *E. coli* strains.

Similar to that reported previously, our findings indicated 5 triclosan-resistance strains showing *fabI* overproduction [31]. Nevertheless, the mutation of *fabI* did not mediate the resistance of *E. coli* to triclosan in our research, which is inconsistent with other reports. This difference can be attributed to the modification in the Gly79Ala and Ala69Thr amino acids in both triclosan-resistant and -susceptible strains in our study [16, 17]. Perhaps, triclosan resistance was mediated by *fabI* and efflux pumps overexpression in our tested strains, rather than by *fabI* mutation. In addition, different pulse types and STs types were recorded in the studied isolates via PFGE and MLST, which suggests no transmission and a clonal dissemination among the tested triclosan-resistant strains.

However, our study also has a limitation. Although we could demonstrate the presence of multiple and cross-resistance between triclosan and antibiotics, the underlying mechanisms for the same remains the focus of our future research.

## Conclusions

We are the first to suggest that *E. coli* isolates can acquire triclosan resistance in only 12 days under the stimulation of subinhibitory concentration of triclosan in vitro and that triclosan exposure can contribute to reduce the susceptibility to clinical antimicrobials, which in turn can accelerate the emergence of cross-resistance or multidrug-resistant bacteria. In addition, we also systematically described that bacteria acquire resistance to triclosan through the overexpression *of fabI* and efflux pumps. In summary, our findings emphasize that the extensive and long-term use of triclosan would warrant improved vigilance with regards to the presence and emergence of multidrug resistance in *E. coli* bacteria present in urine. Moreover, it is important to stress the rational use of triclosan as a control measure against the spread of multiple and cross-resistance.

## Supplementary information

**Additional file 1: Table S1.** The primers used in this study. **Table S2.** The relative gene expression in *E. coli* ATCC 25922 and the field triclosan resistant isolates. **Table S3.** The relative gene expression in the triclosan-resistant induced *Escherichia coli* isolates.

## Data Availability

All data generated or analyzed during this study are included in this published article.

## Abbreviations

ATCC: American Type Cultures Collection
CLSI: Clinical and Laboratory Standards Institute
CCCP: Carbonyl cyanide 3-chlorophenylhydrazone
CDC: Centers for Disease Control and Prevention
*E. coli*: *Escherichia coli*
MICs: Minimal Inhibitory Concentrations
MDR: Multidrug-resistant
MIC_90_: The concentration required to inhibit growth by 90% isolates
MLST: Multilocus sequence typing
MATE: The multidrug and toxic compound extrusion family
MFS: The major facilitator superfamily
LB: Luria Broth
PCR: Polymerase chain reaction
PFGE: Pulse-field gel electrophoresis
RT-qPCR: Quantitative real-time polymerase chain reaction
RND: The resistance nodulation division family
SSIs: Surgical site infections
STs: Sequence types
SMR: The small multi-resistance family
UTI: Urinary tract infection
