# Supplementary material for: The prevalence and mechanism of triclosan resistance in Escherichia coli isolated from urine samples in Wenzhou, China
Source: Antimicrob Resist Infect Control. 2020 Oct 2;9:161. doi: 10.1186/s13756-020-00823-5 (PMC7531082; doi:10.1186/s13756-020-00823-5)
Supplement: Supplementary file 1 — Additional file 1: Table S1. The primers used in this study. Table S2. The relative gene expression in E. coli ATCC 25922 and the field triclosan resistant isolates. Table S3. The relative gene expression in the triclosan-resistant induced Escherichia coli isolates. [file 13756_2020_823_MOESM1_ESM.doc]

**Table S1 The primers used in this study**

| Procedure | Gene name | Strand ^1^ | Primer (5'—3') | Gene classification | Reference ^2^ |
| --- | --- | --- | --- | --- | --- |
| PCR | *fabI* | F  R | ATGGGTTTTCTTTCCGGTAAG  AATGCTGAANCCGCCGTCAA | reductase coding gene | [1] |
| RT-qPCR | *gapA* | F  R | GCTAACCTGAAATGGGACG  GTCCTGGCCAGCATATTTG | reference genes | [1] |
|  | *16S rRNA* | F  R | TCCTACGGGAGGCAGCAG  GGACTACCAGGGTATCTAATCCTGTT |  | [2] |
|  | *fabI* | F  R | GCCGCTCCATGCTGAATCCG  CGCACGTTCGCTTCCAGAGAC | reductase coding gene | [1] |
|  | *norE* | F  R | TCGCAGGACATCAGATTG  CAGACACCCACCATAAGC | NorE efflux pump | [3] |
|  | *ydcT* | F  R | TCCAGGCGGTGCAGTATCAGG  GAGCGTGGCAGGCAGTTCTTC | ABC transporters system | [2] |
|  | *ydcU* | F  R | AAGCCTATGCCTGGACGTTATTGC  GTGAGGAACGCAGTCAGCAGTG |  |  |
|  | *ydcV* | F  R | TGGCAGCACAGCGTAGTGATATTC  ATCGCCAGACCAGTGACAATGC |  |  |
|  | *ydcS* | F  R | AAGCCACGCAGCCACAGTTG  CACGGTAGTGTCATGCCAGTAGC |  |  |
|  | *cysP* | F  R | TGGAACGACCTGGTACGCTCTG  CCATGCCGCAAGGTAGGTATAACG |  |  |
|  | *cysU* | F  R | GTGGTGCTGCCGGAACTATCTC  GTGGTGCTGCCGGAACTATCTC |  |  |
|  | *marA* | F  R | TCATAGCATTTTGGACTGG  TTGCGCGATTTCCGTCAT | Arac family | [1] |
|  | *soxS* | F  R | ATTGACCAGCCGCTTAACAT  ACATAACCCAGGTCCATTG |  |  |
|  | *yihV* | F  R | GGCTATCATCCTCGTCTTCC  GCGTCATCCACCAGTAACC | RND efflux systems | [3] |
|  | *acrB* | F  R | GAAGAGCACGCACCACTACAC  GCAGACGCACGAACAGATAGG |  |  |
|  | *acrD* | F  R | GTGCTGGCAATCCTGTT  TATCGAGGCCGGTCATAT |  | [1] |
|  | *acrF* | F  R | TGGCCATTATYCTGATGA  GTTATCGATACCGTTCATA |  |  |
|  | *mdfA* | F  R | TTTATGCTTTCGGTATTGG  GAGATTAAACAGTCCGTTGC | MdfA efflux pump | [3] |

^1^ F: Forward primer; R: Reverse primer.

^2^ Reference

1. Curiao T, Marchi E, Viti C, Oggioni MR, Baquero F, Martinez JL, Coque TM: **Polymorphic variation in susceptibility and metabolism of triclosan-resistant mutants of Escherichia coli and Klebsiella pneumoniae clinical strains obtained after exposure to biocides and antibiotics**. *Antimicrob Agents Chemother* 2015, **59**(6):3413-3423.

2. Li M, He Y, Sun J, Li J, Bai J, Zhang C: **Chronic Exposure to an Environmentally Relevant Triclosan Concentration Induces Persistent Triclosan Resistance but Reversible Antibiotic Tolerance in Escherichia coli**. *Environ Sci Technol* 2019, **53**(6):3277-3286.

3. Sonbol FI, El-Banna TE, Abd El-Aziz AA, El-Ekhnawy E: **Impact of triclosan adaptation on membrane properties, efflux and antimicrobial resistance of Escherichia coli clinical isolates**. *J Appl Microbiol* 2019, **126**(3):730-739.

**Tables S2 The relative gene expression in *E. coli* ATCC 25922 and the field triclosan** resistant isolates

| Isolate | ATCC 25922 | DC8358 | DC8419 | DC8424 | DC8603 | DC8724 |
| --- | --- | --- | --- | --- | --- | --- |
| Gene name | Relative genes expression (means ± SD) | | | | | |
| *fabI* | 1 | **5.69 ± 0.49** | **25.14 ± 0.42** | **5.11 ± 0.43** | **34.05 ± 0.23** | **41.85 ± 0.59** |
| *norE* | 1 | 0.15 ± 0.02 | 0.56 ± 0.02 | 1.24 ± 0.16 | ND | 0.89 ± 0.06 |
| *ydcT* | 1 | 0.016 ± 0.00 | 0.14 ± 0.01 | 0.56 ± 0.28 | ND | **6.56 ± 0.56** |
| *ydcU* | 1 | 0.06 ± 0.00 | 0.84 ± 0.13 | **4.71 ± 0.13** | ND | **18.25 ± 1.36** |
| *ydcV* | 1 | **5.71 ± 0.68** | **8.74 ± 0.61** | 0.00 ± 0.00 | ND | 1.69 ± 0.18 |
| *ydcS* | 1 | 0.11 ± 0.00 | 1.12 ± 0.09 | **2.80 ± 0.42** | ND | **8.76 ± 0.49** |
| *cysP* | 1 | 0.08 ± 0.01 | 0.02 ± 0.00 | 0.00 ± 0.00 | ND | **3.89 ± 0.20** |
| *cysU* | 1 | 0.07 ± 0.02 | 0.65 ± 0.04 | 0.18 ± 0.05 | ND | 1.17 ± 0.16 |
| *marA* | 1 | 0.11 ± 0.02 | 0.07 ± 0.00 | 0.76 ± 0.04 | ND | 0.21 ± 0.03 |
| *soxS* | 1 | 0.07 ± 0.01 | 0.09 ± 0.00 | 0.98 ± 0.07 | ND | 0.25 ± 0.03 |
| *yihV* | 1 | 0.61 ± 0.01 | **3.57 ± 0.52** | **6.82 ± 0.65** | ND | **2.00 ± 0.03** |
| *acrB* | 1 | 0.24 ± 0.02 | **3.44 ± 0.21** | 1.64 ± 0.10 | ND | 0.19 ± 0.03 |
| *acrD* | 1 | 0.01 ± 0.00 | 0.04 ± 0.00 | **2.63 ± 0.14** | ND | 0.45 ± 0.06 |
| *acrF* | 1 | 0.00 ± 0.00 | 0.00 ± 0.00 | 0.00 ± 0.00 | ND | 0.00 ± 0.00 |
| *mdfA* | 1 | 0.24 ± 0.02 | 0.12 ± 0.00 | **5.13 ± 0.26** | ND | 0.88 ± 0.09 |

ND, Not detected; The values in bold font indicates the gene overexpression, which is the relative expression level increased by 2-fold or greater in comparison to that of the control strain ATCC 25922.

**Tables S3** **The relative gene expression in the triclosan-resistant induced *Escherichia coli* isolates**

| Isolate | DC8361-R | DC8363-R | DC8400-R | DC8413-R | DC8510-R |
| --- | --- | --- | --- | --- | --- |
| Gene name | Relative genes expression (means ± SD) | | | | |
| *norE* | 0.86 ± 0.19 | **2.42 ± 0.07** | **2.02 ± 0.63** | **1.50± 0.29** | 3.24 ± 2.07 |
| *ydcT* | **0.94 ± 0.15** | 0.17 ± 0.05 | 0.53 ± 0.02 | 0.82 ± 0.06 | **0.68 ± 0.12** |
| *ydcU* | **1.64 ± 0.35** | 0.25 ± 0.14 | **0.77 ± 0.03** | 0.11 ± 0.01 | 0.68 ± 0.05 |
| *ydcV* | **1.10 ± 0.29** | 0.31 ± 0.03 | 0.55 ± 0.10 | 0.70 ± 0.07 | **1.12 ± 0.33** |
| *ydcS* | **1.47 ± 0.06** | 0.16 ± 0.07 | **1.10 ± 0.03** | 0.69 ± 0.13 | **1.15 ± 0.22** |
| *cysP* | **1.64 ± 0.16** | 0.78 ± 0.07 | 1.18 ± 0.16 | 1.28 ± 0.08 | 0.79 ± 0.11 |
| *cysU* | 0.37 ± 0.04 | **1.19 ± 0.11** | **0.33 ± 0.03** | 0.69 ± 0.05 | 0.59 ± 0.12 |
| *marA* | 1.15 ± 0.17 | **19.80 ± 1.15** | **3.00 ± 0.16** | **5.21 ± 0.36** | **1.24 ± 0.09** |
| *soxS* | 1.17 ± 0.14 | 2.11 ± 0.19 | **1.49 ± 0.07** | **2.81 ± 1.52** | 1.89 ± 0.23 |
| *yihV* | **7.94 ± 0.17** | 3.92 ± 0.50 | **5.98 ± 0.35** | **7.58 ± 0.95** | **9.09 ± 0.48** |
| *acrB* | **0.49 ± 0.01** | 0.32 ± 0.04 | 0.45 ± 0.03 | **0.58 ± 0.07** | **0.81 ± 0.04** |
| *acrD* | 0.61 ± 0.18 | 1.70 ± 0.21 | **0.75 ± 0.16** | 1.01 ± 0.14 | **1.85 ± 0.32** |
| *acrF* | 0.00 ± 0.00 | 0.00 ± 0.00 | 0.00 ± 0.00 | 0.00 ± 0.00 | 0.00 ± 0.00 |
| *mdfA* | 1.06 ± 0.08 | **2.66 ± 0.30** | **2.19 ± 0.12** | **1.57 ± 0.13** | 1.67 ± 0.13 |

^-R^, triclosan-resistant induced isolates; ^b^ The values in bold font indicates the relative expression levels increased by 2-fold or greater in comparison with those of the parent strains.
